# ﻿Two new species of *Penicillium* (Eurotiales, Aspergillaceae) from China based on morphological and molecular analyses

**DOI:** 10.3897/mycokeys.116.149376

**Published:** 2025-04-25

**Authors:** Rui-Na Liang, Xiang-Hao Lin, Miao-Miao An, Guo-Zhu Zhao

**Affiliations:** 1 College of Biological Sciences and Technology, Beijing Forestry University, Beijing 100083, China Beijing Forestry University Beijing China; 2 National Engineering Research Center of Tree Breeding and Ecological Restoration, Beijing Forestry University, Beijing 100083, China Beijing Forestry University Beijing China

**Keywords:** Aspergillaceae, DNA barcodes, section *Brevicompacta*, section *Lanata-Divaricata*, taxonomy

## Abstract

*Penicillium* is a large and significant genus of fungi, exhibiting widespread distribution across diverse substrates. Ongoing taxonomic and nomenclatural revisions have led to an annual increase in the number of newly described species. This study described two new *Penicillium* species, i.e., *P.lentum* and *P.tibetense*, discovered in China. They have been identified and characterized through morphological examination and both single gene and multigene phylogenetic analyses. Based on these analyses, *P.lentum* was classified within the section Brevicompacta, while *P.tibetense* was placed in the section Lanata-Divaricata. Both species exhibited the morphological features typical of their respective sections. *Penicilliumlentum* is characterized by restricted growth with dense colonies on agar media and predominantly generates terverticillate conidiophores. *Penicilliumtibetense* demonstrates rapid growth on media and has vigorous growth on CYA at 30 °C, producing biverticillate conidiophores. Comprehensive descriptions and detailed illustrations of these new species were presented. A morphological comparison between the new species and their closely related taxa was provided.

## ﻿Introduction

*Penicillium* is widely distributed across various substrates, primarily in soil, as well as in the atmosphere, food, plant tissues, and other environments. Several species possess considerable value for human applications in food production, biocontrol, and biotechnology. For instance, *P.sclerotiorum* exhibits antagonistic activity against certain plant pathogens, demonstrating potential as a biocontrol agent (Jahan et al. 2024). The food industry utilizes *P.nalgiovense* as starter cultures for dry-fermented sausages (Ludemann et al. 2010). The capability of certain species to synthesize pigments has prompted the evaluation of these species for the production of highly stable and safe natural pigments (Morales-Oyervides et al. 2020). Nevertheless, mycotoxins generated by specific species present a significant risk to human and animal health (Nielsen et al. 2017). Notably, patulin exhibits multiple toxicities, including genotoxicity and immunotoxicity, and is predominantly produced by *P.expansum* and *P.griseofulvum* (Bandoh et al. 2009; Puel et al. 2010; Tannous et al. 2014).

Link (1809) introduced the generic name *Penicillium*, which is classified in the family Aspergillaceae. Traditional taxonomy of *Penicillium* primarily relied on morphological characters, including colony diameter, texture, conidial color, and conidiophore branching patterns. However, the variability in morphology has presented substantial challenges in accurately identifying novel species, frequently resulting in the erroneous classification of new isolates under known species (Visagie et al. 2016). Conversely, contemporary taxonomy adopts a polyphasic strategy that incorporates morphological, extrolite, genetic, and multigene phylogenetic data (Visagie et al. 2014). Houbraken et al. (2020) delivered the most comprehensive update on the genus *Penicillium* based on a phylogenetic approach combined with phenotypic, physiologic, and extrolite data. This study recognized 483 species and introduced a novel series classification, which is deemed highly predictive of potential functional traits (Houbraken et al. 2020). Subsequently, Visagie et al. (2024b) applied GCPSR (Genealogical Concordance Phylogenetic Species Recognition) and phylogenetic analyses to reassess the list of *Penicillium* species published up to 31 December 2022, resulting in an updated count of 535 species. An additional 100 species of this genus were described from 1 January 2023 to 31 December 2024 (Ansari et al. 2023; Crous et al. 2023; da Silva et al. 2023; Khuna et al. 2023; Li et al. 2023; Liu et al. 2023; Tan 2023; Tan and Shivas 2023, 2024; Tan et al. 2023, 2024a, 2024b; Wang et al. 2023; Zhang et al. 2023; Araújo et al. 2024; Crous et al. 2024; Liang et al. 2024; Lima et al. 2024; Nóbrega et al. 2024; Song et al. 2024; Visagie et al. 2024a, 2024c; Zhang et al. 2024). The increase in species numbers in recent years indicates the possibility of numerous undiscovered *Penicillium* species, and their biodiversity, ecological functions, and potential for resource development warrant further investigation.

During a comprehensive survey of *Penicillium* biodiversity in China, we found two isolates that could not be classified within existing species. In this paper, we compare these isolates with related species using multi-locus phylogenetic analyses and morphological character assessments. As a result, the isolates are described as species new to science. This study is expected to offer new perspectives on the diversity, function, ecology, and distribution of *Penicillium* members.

## ﻿Materials and methods

### ﻿Isolates

Soil samples were collected from the rhizosphere of plants in the Kangyu Tunnel, Tibet, China, while indoor dust samples were sourced from Beijing Forestry University, Beijing, China. To isolate the fungus, the samples were suspended in sterile water at a ratio of 1:10, vortexed to ensure homogeneity, and then diluted to 10^-4^ concentrations. Each of 100 μL from 10^-2^, 10^-3^, and 10^-4^ dilutions was spread on potato dextrose agar (PDA) and Martin medium with 50 ppm penicillin and 50 ppm streptomycin. The cultures were incubated at 25 °C for 5–7 days. Individual colonies were then picked from the plates and transferred to fresh PDA plates until pure cultures were obtained. Type specimens, preserved as dry cultures, were deposited in the Fungarium (HMAS), Institute of Microbiology, Chinese Academy of Sciences, while ex-type strains, maintained as living cultures, were stored at the China General Microbiological Culture Collection Centre (CGMCC).

### ﻿Morphological studies

Morphological observations of colonies were conducted under strictly standardized conditions, encompassing media preparation, inoculation technique, incubation parameters, and description methods (Visagie et al. 2014). Colony characters and diameters were recorded from cultures grown on Czapek yeast autolysate agar (CYA), malt extract agar (MEA), yeast extract sucrose agar (YES), dichloran 18% glycerol agar (DG18), and creatine sucrose agar (CREA) at 25 °C for 7 days. Additional CYA plates were incubated at 30 and 37 °C. Color names and codes adhered to the book “Color Standards and Color Nomenclature” (Ridgway 1912). Ehrlich reaction was employed to assess the production of indole metabolites; a violet ring observed within ten min was deemed a positive result, while other color changes were interpreted as negative (Lund 1995; Houbraken et al. 2016).

For light microscopic observations, slides were prepared from cultures grown on MEA, and phenol glycerin solution was used as mounting fluid, with cotton blue staining if necessary. In addition, a field emission scanning electron microscope (Hitachi SU8010, Japan) was employed to examine microstructural characteristics. Agar blocks (3–4 mm × 3–4 mm) were fixed in 2.5% v/v glutaraldehyde at 4 °C for 8–12 hr, then washed three times for 10 min each with 0.1M phosphate buffer. Dehydration was performed with a gradient of ethanol (30, 50, 70, 95, and 100% v/v) for 10–20 min per step, followed by replacement with tert-butanol and ultimate vacuum freeze-dried and gold-sprayed for observation (Wei et al. 2024).

### ﻿DNA extraction, sequencing, and phylogenetic analyses

Colonies were cultivated on MEA plates for 5–7 days, and DNA extraction was conducted using the E.Z.N.A.® Fungal DNA Mini Kit (Omega Bio-Tek, Inc., United States). The internal transcribed spacer (ITS), beta-tubulin (*BenA*), calmodulin (*CaM*), and RNA polymerase II second largest subunit (*RPB2*) genes were amplified using primer pairs ITS1/ITS4 (White et al. 1990), Bt2a/Bt2b (Glass and Donaldson 1995), CMD5/CMD6 (Hong et al. 2006), and RPB2-5F/RPB2-7CR (Liu et al. 1999), respectively. Polymerase chain reaction (PCR) amplification followed Visagie et al. (2014). Sequencing reactions were performed by Sangon Biotech (Shanghai) Company Limited, China. DNAMAN software (Lynnon Biosoft) was used for the assembly and trimming of the Sanger chromatograms. Sequences were submitted to GenBank (www.ncbi.nlm.nih.gov).

Sequence similarity searches were conducted using the mega BLAST program of basic local alignment search tool (BLAST) within the NCBI core nucleotide database (core_nt). Comprehensive sequence datasets were compiled containing newly generated sequences alongside reference sequences sourced from GenBank (Table 1). Sequence alignments were performed using the ClustalW algorithm and subsequently manually edited using MEGA 11 (Tamura et al. 2021). The resulting multiple sequence alignments have been deposited in TreeBASE (submission number: 31847) (www.treebase.org). Phylogenetic trees were constructed based on the ITS, *BenA*, *CaM*, and *RPB2* genes as well as the concatenated sequences of the latter three genes. Phylogenetic analyses were conducted using both maximum likelihood (ML) and Bayesian Inference (BI). ML phylogenies were performed using IQtree v. 1.6.12 (Nguyen et al. 2015), including 1000 standard non-parametric bootstrap replicates with the best partition scheme and substitution model selected using ModelFinder (Kalyaanamoorthy et al. 2017). BI phylogenies were run in MrBayes v. 3.2.7 (Ronquist et al. 2012). Best fit models were selected according to the Akaike information criterion (AIC) using MrModeltest v. 2.4 (Nylander 2004). Posterior probabilities (PP) were estimated using Markov Chain Monte Carlo (MCMC) sampling, set to run for 1,000,000 generations with the average standard deviation of split frequencies less than 0.01 as the stopping criterion. In cases where this threshold was not achieved, the run was continued until the condition was met. Additionally, the initial 25% of the generated trees were discarded as burn-in.

**Table 1.** Strains of *Penicillium* used for phylogenetic analyses.

Species	Strain	Substrate and origin	GenBank accession numbers			
			ITS	*BenA*	*CaM*	*RPB2*
*P.abidjanum*	CBS 246.67^T^	Soil, Ivory Coast	GU981582	GU981650	MN969234	JN121469
*P.alagoense*	URM 8086^T^	Leaves of *Miconia* sp., Brazil	MK804503	MK802333	MK802336	MK802338
*P.amphipolaria*	CBS 140997^T^	Soil, Antarctica	KT887872	KT887833	KT887794	MN969177
*P.annulatum*	CBS 135126^T^	Air sample, South Africa	JX091426	JX091514	JX141545	KF296410
*P.araracuaraense*	CBS 113149^T^	Leaf litter, Colombia	GU981597	GU981642	MN969237	KF296414
*P.astrolabium*	CBS 122427^T^	Grapes, Portugal	DQ645804	DQ645793	DQ645808	JN406634
*P.ausonanum*	CBS 148237^T^	Sediment of freshwater stream, Spain	LR655808	LR655809	LR655810	LR655811
*P.austrosinense*	CGMCC 3.18797^T^	Acidic soil, China	KY495007	KY495116	MN969328	KY495061
*P.bialowiezense*	CBS 227.28^T^	Soil under conifers, Poland	EU587315	AY674439	AY484828	JN406604
*P.bissettii*	CBS 140972^T^	Soil from spruce forest, Canada	KT887845	KT887806	KT887767	MN969178
*P.brasilianum*	CBS 253.55^T^	Herbarium exsiccata, Brazil	GU981577	GU981629	MN969239	KF296420
*P.brevicompactum*	NRRL 28139	Stroma of a wood decay fungus, USA	AY484917	DQ645795	AY484825	–
	CV1492	Unknown, South Africa	JX091398	JX091533	JX141574	–
	CBS 257.29^T^	Unknown, Belgium	AY484912	AY674437	AY484813	JN406594
*P.buchwaldii*	CBS 116980	Wheat, United Kingdom	JX313163	JX313181	JX313147	–
	CBS 116935	Wheat, United Kingdom	JX313156	JX313174	JX313140	–
	CBS 116929	Wheat flour, Denmark	JX313152	JX313170	JX313136	–
	CBS 117181^T^	*Hordeumvulgare*, Denmark	JX313164	MN969374	JX313148	JN406637
*P.camponoti*	CBS 140982^T^	Carpenter ants, Canada	KT887855	KT887816	KT887777	MN969179
*P.cataractarum*	CBS 140974^T^	Fallen nuts of *Caryacordiformis*, Canada	KT887847	KT887808	KT887769	MN969180
*P.coffeatum*	CGMCC 3.25152^T^	Soil, China	OQ870815	OR051121	OR051298	OR051466
*P.daleae*	CBS 211.28^T^	Soil under conifer, Poland	GU981583	GU981649	MN969251	KF296427
*P.echinulonalgiovense*	CBS 328.59^T^	Unknown, Japan	GU981587	GU981631	KX961269	KX961301
*P.excelsum*	DTO 357-D7^T^	Brazil nut shell, Brazil	KR815341	KP691061	KR815342	MN969166
	ITAL 7804	Flowers, Brazil	KT749963	KT749959	KT749962	–
*P.expansum*	CBS 325.48^T^	*Malussylvestris*, USA	AY373912	AY674400	DQ911134	JF417427
*P.fengjieense*	CGMCC 3.25157^T^	Soil, China	OQ870765	OR051156	OR051333	OR051489
*P.fennelliae*	CBS 711.68^T^	Soil, Congo	JX313169	MN969382	JX313151	JN406536
*P.flaviroseum*	CGMCC 3.18805^T^	Acidic soil, China	KY495032	KY495141	MN969329	KY495083
*P.fructuariae-cellae*	CBS 145110^T^	Dried fruit of *Vitisvinifera*, Italy	MK039434	KU554679	MK045337	–
*P.globosum*	CBS 144639^T^	Acidic soil, China	KY495014	KY495123	MN969330	KY495067
*P.griseoflavum*	CGMCC 3.18799^T^	Acidic soil, China	KY495011	KY495120	MN969331	KY495064
*P.griseopurpureum*	CBS 406.65^T^	Soil under *Pinus* sp., United Kingdom	KF296408	KF296467	MN969261	KF296431
*P.guaibinense*	CCDCA 11512^T^	Soil, Brazil	MH674389	MH674391	MH674393	–
*P.guangxiense*	CBS 144526^T^	Soil, China	KY494986	KY495095	MN969332	KY495045
*P.hainanense*	CGMCC 3.18798^T^	Acidic soil, China	KY495009	KY495118	MN969333	KY495062
*P.infrabuccalum*	CBS 140983^T^	*Camponotuspennsylvanicus*, Canada	KT887856	KT887817	KT887778	MN969181
*P.jianfenglingense*	CGMCC 3.18802^T^	Acidic soil, China	KY495016	KY495125	MN969334	KY495069
*P.jinyunshanicum*	CGMCC 3.25162^T^	Soil, China	OQ870766	OR051157	OR051334	OR051490
*P.kongii*	AS3.15329^T^	leaf sample of *Cotoneaster* sp., China	KC427191	KC427171	KC427151	–
*P.laevigatum*	CGMCC 3.18801^T^	Acidic soil, China	KY495015	KY495124	MN969335	KY495068
***P.lentum***	**CGMCC 3.28596^T^ = B24**	**Indoor dust, Beijing, China**	**PQ643282**	**PQ519854**	**PQ519855**	**PQ519856**
*P.mariae-crucis*	CBS 271.83^T^	*Secalecereale*, Spain	GU981593	GU981630	MN969275	KF296439
*P.marykayhuntiae*	BRIP 74934a^T^	Soil, Australia	OR271913	OR269446	–	OR269440
*P.neocrassum*	CBS 122428^T^	Grapes, Madeira	DQ645805	DQ645794	DQ645809	JN406633
*P.newtonturnerae*	BRIP 74909a^T^	Soil, Australia	OP903478	OP921964	OP921962	OP921963
*P.ochrochloron*	CBS 357.48^T^	Copper sulphate solution, USA	GU981604	GU981672	MN969280	KF296445
	DTO 189-A6	Unknown, Japan	KC346347	KC346324	KC346341	KC346318
*P.olsonii*	CBS 232.60^T^	*Musa*, France	EU587341	AY674445	DQ658165	JN121464
*P.onobense*	CBS 174.81^T^	Soil, andosol, Spain	GU981575	GU981627	MN969281	KF296447
*P.panissanguineum*	CBS 140989^T^	Soil near termite mound, Tanzania	KT887862	KT887823	KT887784	MN969182
*P.paraherquei*	CBS 338.59^T^	Soil, Japan	AF178511	KF296465	MN969285	KF296449
*P.pauciramulum*	CGMCC 3.25164^T^	Soil, associated with nest of Formicidae, China	OQ870726	OR051111	OR051288	OR051457
*P.pedernalense*	CBS 140770^T^	*Litopenaeusvannamei*, Ecuador	KU255398	KU255396	MN969322	MN969184
*P.penarojense*	CBS 113178^T^	Leaf litter, Colombia	GU981570	GU981646	MN969287	KF296450
*P.piscarium*	CBS 362.48^T^	Cod-liver oil emulsion, Germany	GU981600	GU981668	MN969288	KF296451
*P.pulvillorum*	CBS 280.39^T^	Acidic soil, United Kingdom	AF178517	GU981670	MN969289	KF296452
	CBS 275.83	Rye grain, Spain	GU981601	GU981671	KC346336	KF296423
*P.rolfsii*	CBS 368.48^T^	Fruit of *Ananassativus*, USA	JN617705	GU981667	MN969294	KF296455
*P.roodeplaatense*	DTO 444-C8	Soil, South Africa	OR819195	OR820176	OR820180	OR820186
*P.rotoruae*	CBS 145838^T^	*Pinusradiata* timber stake in ground contact, New Zealand	MN315103	MN315104	MN315102	MT240842
*P.rubriannulatum*	CGMCC 3.18804^T^	Acidic soil, China	KY495029	KY495138	MN969336	KY495080
*P.salamii*	CBS 135391^T^	Salami, Italy	HG514431	HG514437	HG514432	MN969160
*P.simplicissimum*	CBS 372.48^T^	*Secalecereale*, Spain	GU981588	GU981632	MN969297	JN121507
*P.singorense*	CBS 138214^T^	House dust, Thailand	KJ775674	KJ775167	KJ775403	MN969138
*P.skrjabinii*	CBS 439.75^T^	Soil, Russia	GU981576	GU981626	MN969299	EU427252
*P.soliforme*	CGMCC 3.18806^T^	Acidic soil, China	KY495038	KY495147	MN969337	KY495047
	NN072390	Acidic soil, China	KY495019	KY495128	KY494959	KY495072
	NN072399	Acidic soil, China	KY495022	KY495131	KY494962	KY495074
*P.spathulatum*	CBS 117192^T^	Mouldy chestnut (*Castanea* sp.), France	JX313165	MN969400	JX313149	JN406636
*P.spinuliferum*	CBS 144483^T^	Acidic soil, associated with *Litchichinensis*, China	KY495040	KY495149	MN969338	KY495090
*P.stangiae*	URM 8347^T^	Soil, Brazil	MW648590	MW646388	MW646390	MW646392
*P.stolkiae*	CBS 315.67^T^	Soil, South Africa	AF033444	JN617717	AF481135	JN121488
*P.subfuscum*	CBS 147455^T^	Soil, South Africa	MT949907	MT957412	MT957454	MT957480
*P.subrubescens*	CBS 132785^T^	Soil of *Helianthustuberosus* field, Finland	KC346350	KC346327	KC346330	KC346306
*P.subrutilans*	CGMCC 3.25174^T^	Soil, China	OQ870816	OR051137	OR051314	OR051479
*P.svalbardense*	CBS 122416^T^	Glacial ice, Svalbard	GU981603	DQ486644	KC346338	KF296457
*P.taii*	CGMCC 3.25176^T^	Soil, China	OQ870778	OR051170	OR051347	OR051496
*P.tanzanicum*	CBS 140968^T^	Soil near termite mound, Tanzania	KT887841	KT887802	KT887763	MN969183
*P.terrarumae*	CBS 131811^T^	Soil contaminated by heavy metals, China	MN431397	KX650295	MN969323	MN969185
	CS23-08	Unknown, China	OQ870751	OR051141	OR051318	OR051481
*P.tularense*	CBS 430.69^T^	Soil under *Pinusponderosa* and *Quercuskelloggii*, USA	AF033487	KC427175	JX313135	JN121516
	CBS 431.69	Soil under *Pinusponderosa* and *Quercuskelloggii*, USA	JX313167	AY674433	JX313134	–
*P.vanderhammenii*	CBS 126216^T^	Leaf litter, Colombia	GU981574	GU981647	MN969308	KF296458
*P.vasconiae*	CBS 339.79^T^	Soil, Spain	GU981599	GU981653	MN969309	MN969144
*P.vickeryae*	BRIP 72552a^T^	Soil, Australia	OP903479	OP921966	–	OP921965
*P.viridissimum*	CGMCC 3.18796^T^	Acidic soil, China	KY495004	KY495113	MN969339	KY495059
*P.wotroi*	CBS 118171^T^	Leaf litter, Colombia	GU981591	GU981637	MN969313	KF296460
***P.tibetense***	**CGMCC 3.28597^T^ = XZ5-3**	**Rhizosphere soil, Tibet, China**	**PQ643284**	**PQ519857**	**PQ519858**	**PQ519859**
*P.yuyongnianii*	CGMCC 3.25187^T^	Soil, China	OQ870820	OR051175	OR051352	OR051499
*P.zonatum*	CBS 992.72^T^	Soil, USA	GU981581	GU981651	MN969315	KF296461

## ﻿Results

### ﻿Morphology

Two novel species, *Penicilliumlentum* and *P.tibetense*, were introduced within the sections *Brevicompacta* and *Lanata-Divaricata*, respectively, based on comprehensive phylogenetic analyses. General morphological characteristics and ecological information for the species included in these sections are provided in Table 2. Both newly described species exhibited morphological traits consistent with their respective sections. Specifically, *P.lentum* displayed limited growth with dense colonies on agar media and primarily produced terverticillate conidiophores. In contrast, *P.tibetense* demonstrated rapid growth on agar media, particularly exhibiting robust development on CYA at 30 °C, and predominantly formed biverticillate conidiophores. The morphological features of the new species and their closely related species are summarized in Table 3.

**Table 2.** Morphological and ecological data pertaining to the sections of the new species in this study.

Section	Morphology	Ecology	References
*Brevicompacta*	Colonies restricted (occasionally moderately fast), texture velutinous; conidiophores terverticillate or multiramulate branched with wide stipes, smooth-walled.	Mainly soil and foods, also on plant leaves and rotting wood.	(Houbraken and Samson 2011; Frisvad et al. 2013; Wang and Wang 2013; Houbraken et al. 2020)
*Lanata-Divaricata*	Colonies grow rapidly, occasionally moderately fast; conidiophores monoverticillate, biverticillate or divaricate, occasionally terverticillate.	Commonly found in soil, also on rotting leaf litter and vegetable.	(Houbraken and Samson 2011; Houbraken et al. 2020)

**Table 3.** Morphological features of new species and their closely related taxa.

Species	Growth rates (mm)			Conidiophores branching	Cleistothecia /sclerotia	Conidia			Acid production on CREA
	CYA	CYA 30 °C	CYA 37 °C			Size	Shape	Roughening	
***P.lentum***	7–10	No growth	No growth	Terverticillate, sometimes biverticillate	Absent	2–3 × 1.5–2.5 μm	Broadly ellipsoidal	Smooth	Absent
*P.tularense* ^a^	n.a.	n.a.	n.a.	Asymmetric and divaricate	Cleistothecia	2.2–2.6 μm	Globose to subglobose	Smooth	n.a.
***P.tibetense***	42–50	42–52	21–27	Biverticillate	Absent	1.5–3 μm	Globose to subglobose	Finely rough	Absent
*P.excelsum* ^b^	35–50	n.a.	8–22	Biverticillate, sometimes terverticillate	Absent	4–5 × 2–3.2 μm	Ellipsoidal	Smooth	Absent

^a^Description based on Paden (1971), ^b^Description based on Taniwaki et al. (2016).

### ﻿Phylogenetic analyses

A BLAST search revealed that strain CGMCC 3.28596 is most closely related to *Penicilliumtularense* (Identities: ITS: 97.52%, *BenA*: 81.13%, *CaM*: 84.91%, *RPB2*: 91.00%) within section Brevicompacta, and strain CGMCC 3.28597 exhibits the highest similarity to *P.excelsum* (Identities: ITS: 98.64%, *BenA*: 94.37%, *CaM*: 89.66%, *RPB2*: 94.84%) within section Lanata-Divaricata.

#### ﻿Section Brevicompacta

**Figure 1.**
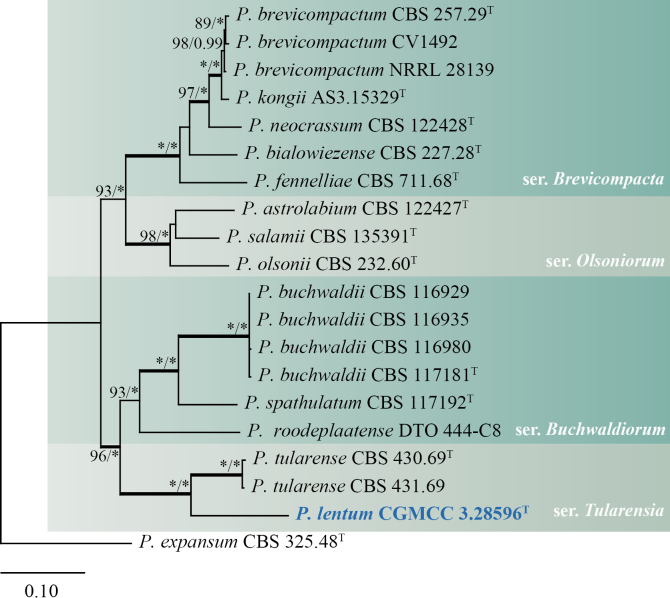
ML tree based on the concatenated data set (*BenA*, *CaM*, and *RPB2*) of section Brevicompacta. *Penicilliumexpansum* CBS 325.48^T^ was designated as the outgroup. Nodes display bootstrap values (bs) exceeding 70% or posterior probabilities (pp) greater than 0.95. Branches with bs of 95% or higher and pp of 1.00 are depicted in bold. The strain described as the new species *P.lentum* is indicated with blue text. * Indicates bs = 100% or pp = 1.00, ^T^ = ex-type strain.

**Figure 2.**
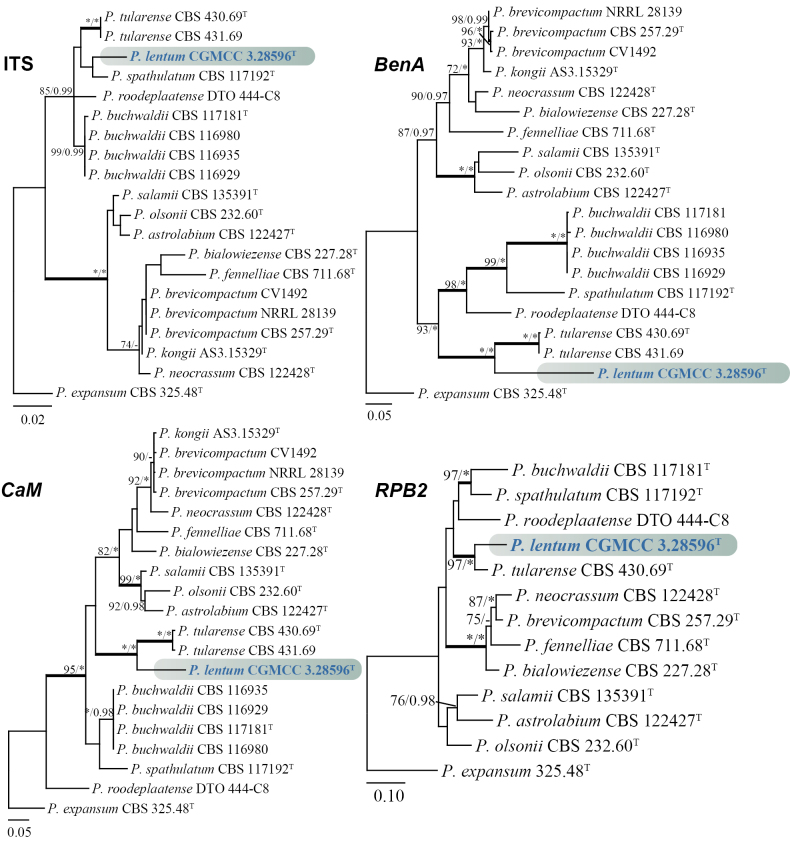
ML trees for section Brevicompacta based on ITS, *BenA*, *CaM*, and *RPB2*. *Penicilliumexpansum* CBS 325.48^T^ was designated as the outgroup. Nodes display bootstrap values (bs) exceeding 70% or posterior probabilities (pp) greater than 0.95. Branches with bs of 95% or higher and pp of 1.00 are depicted in bold. The strain described as the new species *P.lentum* is indicated with blue text. * Indicates bs = 100% or pp = 1.00, ^T^ = ex-type strain.

The analyses of the concatenated dataset (*BenA*, *CaM*, and *RPB2*) comprised 20 predominantly ex-type strains, each with a total sequence length of 1876 bp (*BenA*: 469 bp, *CaM*: 512 bp, *RPB2*: 895 bp). Phylogenetic analyses divided section Brevicompacta into four distinct clades, with the new species *Penicilliumlentum* forming a robustly supported clade alongside *P.tularense* (100% bs, 1.00 pp) (Fig. 1). In the phylogenetic analyses of individual genes, the new species, together with *P.tularense*, consistently formed a well-supported clade, mostly with high support values (>97% bs, 1.00 pp), except for ITS (Fig. 2).

#### ﻿Section Lanata-Divaricata

**Figure 3.**
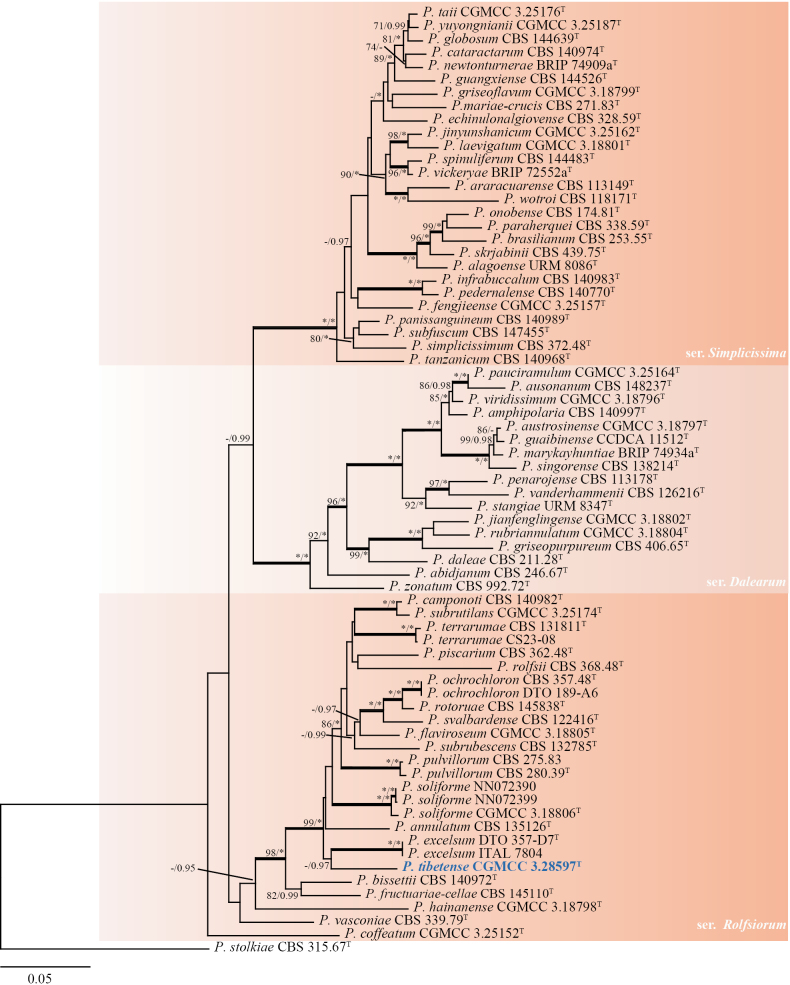
ML tree based on the concatenated data set (*BenA*, *CaM*, and *RPB2*) of section Lanata-Divaricata (series *Simplicissima*, *Dalearum*, and *Rolfsiorum*). *Penicilliumstolkiae* CBS 315.67^T^ was designated as the outgroup. Nodes display bootstrap values (bs) exceeding 70% or posterior probabilities (pp) greater than 0.95. Branches with bs of 95% or higher and pp of 1.00 are depicted in bold. The strain described as the new species *P.tibetense* is indicated with blue text. * Indicates bs = 100% or pp = 1.00, ^T^ = ex-type strain.

**Figure 4.**
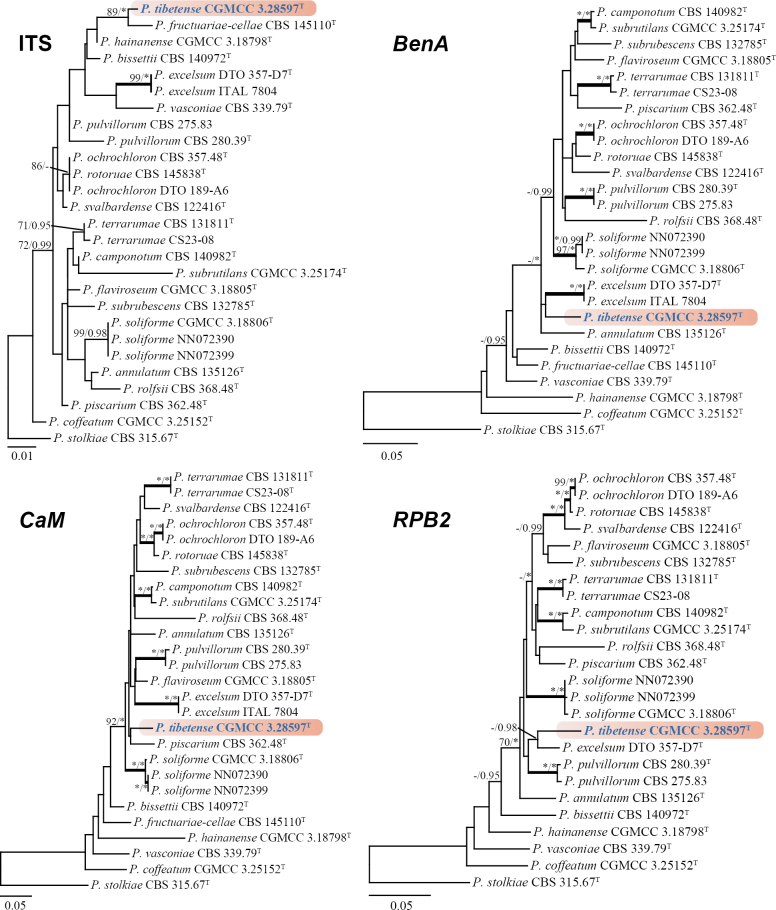
ML trees for section Lanata-Divaricata series *Rolfsiorum* based on ITS, *BenA*, *CaM*, and *RPB2*. *Penicilliumstolkiae* CBS 315.67^T^ was designated as the outgroup. Nodes display bootstrap values (bs) exceeding 70% or posterior probabilities (pp) greater than 0.95. Branches with bs of 95% or higher and pp of 1.00 are depicted in bold. The strain described as the new species *P.tibetense* is indicated with blue text. * Indicates bs = 100% or pp = 1.00, ^T^ = ex-type strain.

In this section, we selected the series *Simplicissima*, *Dalearum*, and *Rolfsiorum*, comprising 71 predominantly ex-type strains, for phylogenetic analyses based on the concatenated dataset totaling 1876 bp (*BenA*: 504 bp, *CaM*: 617 bp, *RPB2*: 755 bp). The resulting phylogenies revealed that *Penicilliumtibetense* is closely related to *P.excelsum* (64% bs, 0.97 pp; not depicted in Fig. 3). However, the significant evolutionary divergence observed supports the recognition of *P.tibetense* as a distinct species (Fig. 3). Phylogenetic analyses of individual genes within series *Rolfsiorum* demonstrated generally weak clustering support, with variations observed among the ITS, *BenA*, *CaM*, and *RPB2* datasets. Furthermore, *P.ochrochloron* and *P.rotoruae* share identical ITS sequences, making them indistinguishable through ITS phylogeny alone (Fig. 4).

## ﻿Taxonomy

### ﻿ Penicillium lentum

Taxon classificationFungiEurotialesAspergillaceae

R.N. Liang & G.Z. Zhao sp. nov.

FB073BBB-D21A-5B35-821E-2C454AEB9EA2

857346

Fig. 5

#### Infrageneric classification.

Subgenus Penicillium, section Brevicompacta, series *Tularensia*.

**Figure 5.**
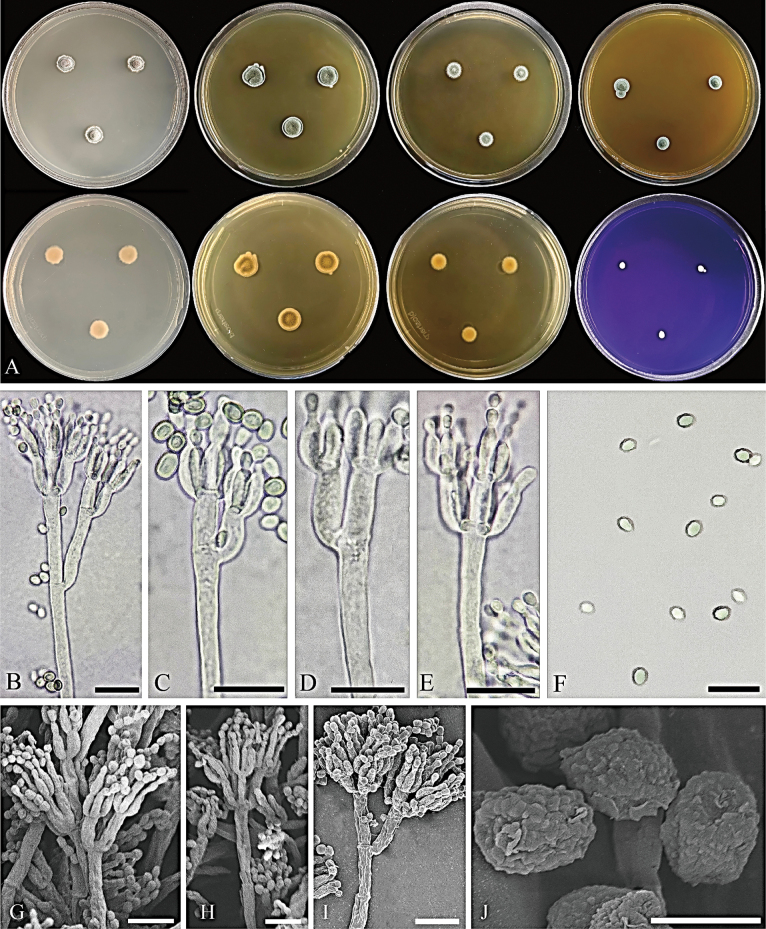
*Penicilliumlentum*CGMCC 3.28596. **A** Colonies on medium at 25 °C for 7d (left to right, top row: CYA, YES, DG18, MEA obverse; second row: CYA reverse, YES reverse, DG18 reverse, CREA obverse) **B–E** conidiophores **F** conidia **G–I** SEM micrograph of conidiophores **J** SEM micrograph of conidia. Scale bars: 10 μm (**B–I**); 2 μm (**J**).

#### Etymology.

The specific epithet “*lentum*” is derived from lentus (Latin), reflecting the slow growth rate characteristic of this species.

#### Type.

China • Beijing, Haidian District, Beijing Forestry University, 40°0'20"N, 116°20'51"E, from indoor dust, 1 February 2024, collected by G.Z. Zhao, B24 (holotype HMAS 353385, dried culture; culture ex-type CGMCC 3.28596).

#### Colony diameter after 7 d (mm).

CYA 7–10; CYA 30 °C, 37 °C no growth; MEA 6–9; YES 9–13; DG18 7–11; CREA 3.5–5.

#### Colony characteristics (7 d).

CYA at 25 °C: Colonies deep, raised at center, margins low, narrow, irregular; mycelium white; texture velutinous, floccose areas present; sporulation moderate to good, conidia antique green (R. Pl. VI); exudate clear; reverse capucine buff (R. Pl. III); soluble pigment absent. MEA at 25 °C: Colonies deep, raised at center, margins low, narrow, entire; mycelium white; texture velutinous, floccose areas present; sporulation moderate to good, conidia celandine green (R. Pl. XLVII) to deep turtle green (R. Pl. XXXII); exudate clear; reverse light orange-yellow (R. Pl. III); soluble pigment absent. YES at 25 °C: Colonies deep, radially and concentrically sulcate, raised at center, margins low, narrow, entire; mycelium white; texture velutinous and fasciculate; sporulation good to strong, conidia glaucous-green (R. Pl. XXXIII); exudate absent; reverse cinnamon (R. Pl. XXIX); soluble pigment absent. DG18 at 25 °C: Colonies low, plane, margins low, wide, entire; mycelium white; texture velutinous and fasciculate; sporulation good, conidia bluish gray-green (R. Pl. XLII); exudate absent; reverse antimony yellow (R. Pl. XV); soluble pigment absent. CREA at 25 °C: Weak growth, no acid production. Ehrlich reaction negative.

#### Micromorphology.

***Conidiophores*** biverticillate to terverticillate; ***stipes*** smooth-walled, 70–236.5 × 2.5–4.5 μm; ***rami*** two when present, 6.5–18 × 2–4 μm; ***metulae*** divergent, 2–4 per branch/ramus, 4.0–13.0 × 2.5–4.5 μm; ***phialides*** ampulliform, 3–8 per metula, 4.5–8.0 × 2–3 μm; ***conidia*** broadly ellipsoidal, smooth-walled, 2–3 × 1.5–2.5 μm.

#### Notes.

*Penicilliumlentum* belongs to section Brevicompacta and is most closely related to *P.tularense* (Fig. 1). *Penicilliumtularense* produces light brown to pale tan cleistothecia, which are not found in the new species (Paden 1971). Additionally, *P.lentum* has broadly ellipsoidal conidia, while *P.tularense* produces globose to subglobose conidia (Table 3).

### ﻿ Penicillium tibetense

Taxon classificationFungiEurotialesAspergillaceae

R.N. Liang & G.Z. Zhao sp. nov.

F6FB447C-7560-5628-946F-5078538CE69E

857347

Fig. 6

#### Infrageneric classification.

Subgenus Aspergilloides, section Lanata-Divaricata, series *Rolfsiorum*.

**Figure 6.**
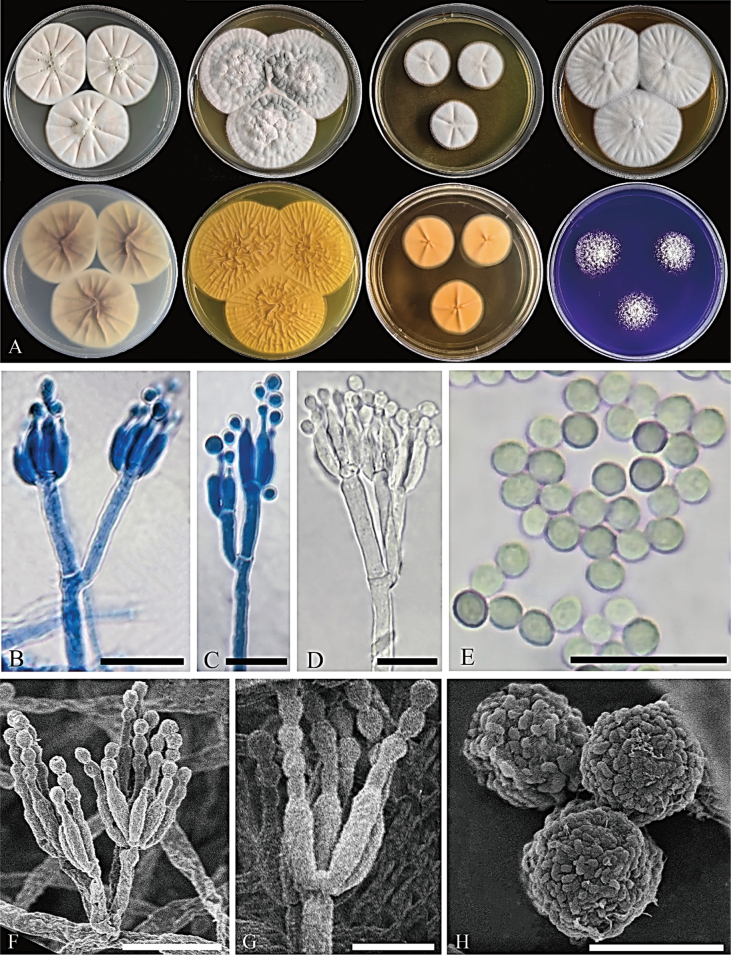
*Penicilliumtibetense*CGMCC 3.28597. **A** Colonies on medium at 25 °C for 7d (left to right, top row: CYA, YES, DG18, MEA obverse; second row: CYA reverse, YES reverse, DG18 reverse, CREA obverse) **B–D** conidiophores **E** conidia **F, G** SEM micrograph of conidiophores **H** SEM micrograph of conidia. Scale bars: 10 μm (**B–G**); 2 μm (**H**).

#### Etymology.

The specific epithet “*tibetense*” denotes the geographical origin of the species, indicating its discovery in Tibet.

#### Type.

China • Tibet, Changdu City, Basu County, Kangyu Tunnel, 30°33'53"N, 96°15'25"E, from rhizosphere soil of grasses, 19 July 2023, collected by X.W. Peng, XZ5-3 (holotype HMAS 353386, dried culture; culture ex-type CGMCC 3.28597).

#### Colony diameter after 7 d (mm).

CYA 42–50; CYA 30 °C 42–52; CYA 37 °C 21–27; MEA 48–52; YES 46–52; DG18 20–26; CREA 24–26.

#### Colony characteristics (7 d).

CYA at 25 °C: Colonies low to moderately deep, radially sulcate, margins low, narrow, entire; mycelium white; texture floccose; sporulation moderate, conidia livid pink (R. Pl. XXVII); exudate clear; reverse light purple-drab (R. Pl. XLV) to avellaneous (R. Pl. XL); soluble pigment absent. CYA at 30 °C: Colonies low to moderately deep, radially sulcate, margins low, narrow, entire; mycelium white; texture floccose; sporulation moderate, conidia livid pink (R. Pl. XXVII); exudate clear; reverse brownish vinaceous (R. Pl. XXXIX); soluble pigment absent. CYA at 37 °C: Colonies moderately deep, radially sulcate, margins low, narrow, entire; mycelium white; texture floccose; sporulation sparse, conidia livid pink (R. Pl. XXVII); exudate clear; reverse light buff (R. Pl. XV); soluble pigment absent. MEA at 25 °C: Colonies low to moderately deep, radially sulcate, margins low, narrow, entire; mycelium white; texture floccose; sporulation sparse to moderate, conidia pale brownish vinaceous (R. Pl. XXXIX); exudate clear; reverse antimony yellow (R. Pl. XV); soluble pigment absent. YES at 25 °C: Colonies moderately deep, randomly sulcate, margins low, wide, entire; mycelium white; texture floccose; sporulation moderate, conidia antique green (R. Pl. VI); exudate clear; reverse antimony yellow (R. Pl. XV); soluble pigment absent. DG18 at 25 °C: Colonies low, radially sulcate, margins low, wide, entire; mycelium white; texture floccose; sporulation sparse, conidia ecru-drab (R. Pl. XLVI); exudate absent; reverse orange-pink (R. Pl. II); soluble pigment absent. CREA at 25 °C: Strong growth, no acid production. Ehrlich reaction negative.

#### Micromorphology.

***Conidiophores*** biverticillate; ***stipes*** finely rough-walled, 27–364.5 × 2–3 μm; ***metulae*** appressed to divergent, 2–4 per stipe, 8–15 × 1.5–3 μm; ***phialides*** ampulliform to cylindrical, 2–6 per metula, 5–10.5 × 1.5–3 μm; ***conidia*** globose to subglobose, finely rough-walled, 1.5–3 μm diam.

#### Notes.

*Penicilliumtibetense* is classified in section Lanata-Divaricata and exhibits a close phylogenetic relationship to *P.excelsum* (Fig. 3). This novel species generates globose to subglobose, finely rough-walled conidia that distinguish it from *P.excelsum* (Table 3). Additionally, *P.tibetense* demonstrates more robust growth on CYA at 37 °C compared to *P.excelsum* (21–27 mm vs. 8–22 mm) (Taniwaki et al. 2016).

## ﻿Discussion

*Penicillium*, a ubiquitous and diverse fungal genus, plays pivotal roles in natural ecosystems while maintaining substantial economic importance and significant relevance to human affairs. The recent rapid increase in newly described species within this genus suggests that numerous taxa remain undiscovered. Given the extensive biotechnological applications of *Penicillium* species, accurate taxonomic identification is paramount, necessitating comprehensive species delineation through polyphasic approaches. In the present study, we introduced two novel species: one belonging to section Brevicompacta and the other to section Lanata-Divaricata.

Section Brevicompacta currently comprises 15 species distributed across four series (Visagie et al. 2024a, 2024b) and is represented in our findings by the newly described *P.lentum* sp. nov. This species, classified within series *Tularensia*, is characterized by predominantly terverticillate conidiophores and demonstrates a close relationship with other members of section Brevicompacta (Table 2). Section Lanata-Divaricata is characterized by its remarkable species diversity and rapid colony growth, primarily comprising soil-inhabiting species, with over 90 taxa currently recognized across five series (Houbraken et al. 2020; Visagie et al. 2024b). The newly identified *Penicilliumtibetense* assigned to series *Rolfsiorum* exhibits characteristic rapidly expanding colonies and produces biverticillate conidiophores, consistent with the morphological features typical of this series. Members of section Lanata-Divaricata are ecologically significant as decomposers of organic matter (Lichtner et al. 2022), with notable biotechnological potential exemplified by *P.subrubescens*, which has demonstrated efficient inulinase production (Mansouri et al. 2013).

Phylogenetic analyses of section Brevicompacta demonstrated that our strain *P.lentum* formed a well-supported clade with its closest relative, *P.tularense* (Fig. 1), a relationship corroborated by shared morphological characteristics such as conidiophore branching patterns and growth rates. However, our strain could be clearly distinguished from *P.tularense* based on distinct phenotypic features, including the presence or absence of cleistothecia and differences in conidial morphology (Table 3). The phylogenetic relationships within section Lanata-Divaricata remain unresolved, primarily due to the poor support values in certain clades (Houbraken et al. 2020), exemplified by a clade comprising *P.camponoti*, *P.piscarium*, *P.rolfsii*, *P.subrutilans*, and *P.terrarumae* (Fig. 3), which highlights the persistent challenges in resolving certain taxonomic groups even with multigene phylogenetic approaches.

To address these limitations, we recommend expanding the taxonomic sampling to include strains from diverse geographical origins and ecological niches. This strategy would not only generate additional reference sequences but also facilitate the discovery of novel species and the detection of infraspecific variation (Visagie and Houbraken 2020). Furthermore, sequencing additional gene regions represents a promising approach to enhance phylogenetic resolution (Visagie et al. 2021). The rapid development of high-throughput sequencing technologies has resulted in an accelerated increase in genomic data availability (Kapli et al. 2020), positioning phylogenomics as an essential tool in modern fungal taxonomy and systematics. By leveraging genome-scale data, phylogenomics is poised to overcome the limitations of single gene or multigene analyses, providing robust statistical support for clade resolution and enabling the reconstruction of a highly resolved fungal tree of life (Burki et al. 2020; Zhou and May 2023). These advancements underscore the transformative potential of phylogenomics in addressing long-standing taxonomic challenges and refining our understanding of fungal evolutionary relationships.

## Supplementary Material

XML Treatment for Penicillium lentum

XML Treatment for Penicillium tibetense

## ﻿Additional information

### Data availability

All of the data that support the findings of this study are available in the main text. All sequences generated in this study have been submitted to GenBank.
